# How We Treat Fever and Hypotension in Pediatric Hematopoietic Cell Transplant Patients

**DOI:** 10.3389/fonc.2020.581447

**Published:** 2020-09-16

**Authors:** Matt S. Zinter, Christopher C. Dvorak, Jeffery J. Auletta

**Affiliations:** ^1^Division of Critical Care Medicine, UCSF Benioff Children’s Hospital, University of California, San Francisco, San Francisco, CA, United States; ^2^Division of Allergy, Immunology, and Blood and Marrow Transplantation, UCSF Benioff Children’s Hospital, University of California, San Francisco, San Francisco, CA, United States; ^3^Division of Hematology, Oncology, Blood and Marrow Transplantation, Nationwide Children’s Hospital, Columbus, OH, United States; ^4^Division of Infectious Diseases, Nationwide Children’s Hospital, Columbus, OH, United States

**Keywords:** bone marrow transplant, fever, hypotension, infection, sepsis

## Abstract

Pediatric allogeneic hematopoietic cell transplant (HCT) survival is limited by the development of post-transplant infections. In this overview, we discuss a clinical approach to the prompt recognition and treatment of fever and hypotension in pediatric HCT patients. Special attention is paid to individualized hemodynamic resuscitation, thorough diagnostic testing, novel anti-pathogen therapies, and the multimodal support required for recovery. We present three case vignettes that illustrate the complexities of post-HCT sepsis and highlight best practices that contribute to optimal transplant survival in children.

## Introduction

**FIGURE 1:**
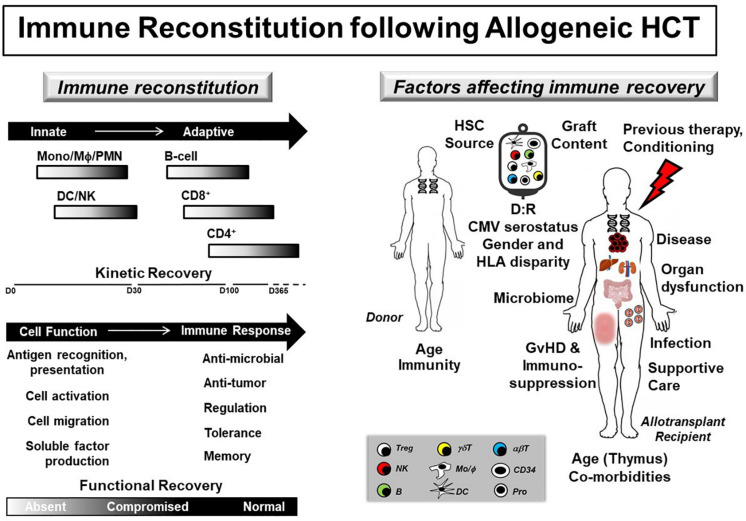
Immune reconstitution following allogeneic HCT. **(Left panel)** Immune reconstitution gradually recovers following allogeneic HCT with donor-derived innate immune cell numbers and function preceding adaptive immunity. **(Right panel)** Kinetic and functional immune recovery in the allogeneic HCT recipient are affected by donor (age, immune function), graft (HSC source, cell content), and recipient (age, presence of co-morbidities and thymic involution or damage, underlying disease and associated previous therapy, type of conditioning regimen, organ dysfunction, changes in microbiome, occurrence of GvHD and associated use of immunosuppression, infection and use of supportive care pharmaceutical agents) factors. In addition, disparities in donor (D) and recipient (R) CMV serostatus, gender and HLA matching also affect donor-derived immune recovery. Defects and delays in immune reconstitution strongly associate with decreased overall survival and increased transplant-related morbidity and mortality. αβT, alpha beta T cell; B, B-cell; CMV, cytomegalovirus; DC, dendritic cell; γδT, gamma delta T cell; GvHD, graft-versus-host disease; HLA, human leukocyte antigen; HSC, hematopoietic stem cell; MΦ, macrophage; Mono, monocyte; NK, natural killer; PMN, polymorphonuclear cell; Pro, progenitor cell; T_reg_, T regulatory cell.

Infections following allogeneic hematopoietic cell transplantation (HCT) are common and negatively impact transplant outcomes (1). Defects in donor-derived immune reconstitution (IR) increase infection risk throughout the peri- and post-transplant periods (Figure 1). As a result, pediatric HCT patients are at significant risk for developing infection and subsequent sepsis. The spectrum of pediatric sepsis can be divided into systemic inflammatory response (SIRS), sepsis, severe sepsis, and septic shock (2). SIRS is defined by the presence of two of the following four criteria, one of which must be abnormal temperature or leukocyte count: core temperature >38.5°C or <36°C; depressed or elevated leukocyte for age; and tachycardia or tachypnea per standard definitions (3). Sepsis is defined by SIRS with a suspected or proven underlying infection, while severe sepsis requires cardiovascular organ dysfunction, acute respiratory distress syndrome or two other organ system involvement (3). Lastly, septic shock is cardiovascular dysfunction refractory to initial fluid resuscitation.

Hematopoietic cell transplantation patients with severe sepsis have a 4-fold increase in hospital mortality versus non-HCT children with severe sepsis (4). Therefore, early recognition and implementation of therapeutic strategies are critical to improving outcomes for severe sepsis, especially in high-risk patient populations (1). To this end, key emerging clinical issues for an allogeneic HCT patient presenting with fever and hypotension include: (1) practical use of new diagnostics for diagnosing infection and sepsis, (2) consideration of multi-drug resistant (MDR) pathogens in certain clinical settings, (3) judicious use of empiric antimicrobial therapies to prevent and to treat MDR pathogens as well as the appropriate duration and de-escalation in antimicrobial therapy, and (4) intensive care support for the septic HCT patient in the context of severe organ dysfunction. We discuss these four aspects in the following clinical vignettes.

## Clinical Vignette #1

### Initial Clinical Presentation

A 5-year-old male with FLT3 internal tandem duplication (ITD)-positive acute myeloid leukemia (AML) in first complete remission (CR1) develops fever and hypotension 12 days following matched unrelated donor (MUD) bone marrow transplant (BMT) with targeted busulfan/cyclophosphamide/antithymocyte globulin (ATG) conditioning. He receives methotrexate (MTX) and tacrolimus for graft-versus-host disease (GvHD) prophylaxis and levofloxacin, acyclovir, fluconazole, and pentamidine for antimicrobial prophylaxis. Vital signs are as follows: Temperature 40°C despite acetaminophen, RR 28, HR 136, BP 80/34, SpO_2_ 97% on room air. Physical exam demonstrates a pale, irritable, but responsive child with tachycardia and oral mucositis. His double-lumen tunneled catheter site has mild erythema at the insertion site but is otherwise without discharge or tenderness. Laboratory studies from earlier in the day are as follows: WBC < 0.1 × 10^3^/μL (reference 6–17 × 10^3^/μL), Hgb 8.2 g/dL (11.5–13.5 g/dL), platelets 15 × 10^3^/μL (140–440 × 10^3^/μL), Cr 0.4 mg/dL (0.2–0.4 mg/dL), ALT 83 U/L (<40 U/L), AST 125 U/L (<50 U/L), total bilirubin 1.2 mg/dL (0.1–1.0 mg/dL), and direct bilirubin 0.2 mg/dL (<0.6 mg/dL).

### What Additional Clinical and Laboratory Information Would Be Useful at This Time?

Sepsis is a clinical diagnosis, and this patient meets diagnostic criteria for sepsis. Sepsis and septic shock must be the top diagnostic consideration in HCT recipients presenting with SIRS criteria. Other potential non-infectious etiologies include engraftment syndrome, anaphylaxis, transfusion reaction and hemophagocytic lymphohistiocytosis (HLH). These potential etiologies should be considered in parallel with sepsis, but their consideration should not delay sepsis treatment. Rapid staging of multi-organ injury (MOI) can help set the tempo of resuscitation and guide downstream management. Further workup should include a blood gas with lactate and procalcitonin as sepsis biomarkers (5, 6), comprehensive metabolic panel to assess for progressive renal and hepatic injury, coagulation studies, fibrinogen, and d-dimers to assess for disseminated intravascular coagulopathy (DIC) and repeat CBC to guide potential need for transfusion. Physical exam looking for a potential source of infection should be performed promptly. Blood cultures from all lumens of indwelling central venous catheters (CVCs) should be obtained but should not postpone empiric antibiotic administration.

#### Take Home Message

Sepsis is a clinical diagnosis and a low-level of clinical suspicion is warranted in an immunocompromised patient in whom multiple infectious etiologies are possible.

### What Are the Most Important First Steps in Clinical Management?

Recognition of neutropenic sepsis should be followed by empiric antibiotic coverage (Table 1) and aggressive fluid resuscitation within the first hour of clinical presentation (7). Prompt empiric antimicrobial therapy in the neutropenic patient should include a broad-spectrum agent with activity against *Pseudomonas* spp. such as 3rd- or 4th-generation cephalosporins or combination β-lactam and β-lactamase inhibitors (e.g., piperacillin-tazobactam) (8). If mucosal barrier injury (MBI) is present or suspected, anaerobic coverage should also be provided with metronidazole, particularly if an extended-spectrum cephalosporin is used as empiric therapy. Methicillin-resistant *Staphylococcus aureus* (MRSA) coverage with a glycopeptide would be added based on patient risk-factors, including clinical instability or evidence of skin or soft tissue infection (8). In addition, pediatric HCT patients receiving levofloxacin prophylaxis are at risk for bacteremia from viridans group *Streptococcus* (9). Therefore, the current patient should receive empiric vancomycin.

**TABLE 1:** Considerations for infectious pathogens associated with sepsis in pediatric allogeneic hematopoietic cell transplant recipients.

**Gram-positive bacteria**	**Yeasts**
*Clostridium septicum*	Candida albicans*
*Enterococcus faecalis*	*Candida krusei*
*Enterococcus faecium*	Candida glabrata*
*Enterococcus* spp., *vancomycin resistant*	*Candida lusitaniae*
Staphylococcus, coagulase negative*	Candida parapsilosis*
Staphylococcus aureus, methicillin resistant*	*Candida tropicalis*
Staphylococcus aureus, methicillin sensitive*	*Cryptococcus neoformans*
Streptococcus, Group A*	*Histoplasma capsulatum*
*Streptococcus, Group B*	
Streptococcus pneumoniae*	
Viridans Streptococcus*	

**Gram-negative bacteria**	**Molds**

*Acinetobacter* spp.	*Aspergillus flavus*
*Bacteroides* spp.	Aspergillus fumigatus*
*Capnocytophaga* spp.	*Aspergillus niger*
*Citrobacter freundii*	*Aspergillus terreus*
Escherichia coli*	*Fusarium* spp.*
Enterobacter cloacae*	*Scedosporidium* spp.
*Fusobacterium* spp.	Zygomycetes*
*Klebsiella oxytoca*	
	
Klebsiella pneumonia*	**Viruses**
Pseudomonas aeruginosa*	Adenovirus*
*Pseudomonas* spp.	Cytomegalovirus*
*Serratia marcescens*	*Herpes simplex virus*
*Stenotrophomonas maltophilia*	*Varicella zoster virus*

spp., species. *Most common pathogens within indicated class causing sepsis.

Fluid resuscitation should focus on prompt restoration of intravascular volume status using available crystalloids (10). Data supporting the use of balanced (PlasmaLyte, Ringer’s lactate, RL) vs. unbalanced (normal saline, NS) crystalloid solutions in pediatric sepsis are equivocal at this time (11). Therefore, fluid resuscitation should be initiated without delay and up to 60 mL/kg of IV fluid should be given in the first hour, with frequent clinical assessment for resolution of tachycardia and impaired perfusion. Red blood cell (RBC) transfusion may be considered; but in the absence of severe anemia or bleeding, should not be administered in lieu of the first 60 mL/kg of crystalloid resuscitation (12). Platelet transfusions may also be considered, particularly if the patient has DIC either with active mucosal bleeding with a platelet count ≤50 × 10^3^/μL or without active bleeding and a platelet count ≤20 × 10^3^/μL (13).

#### Take Home Message

Early empiric antibiotics and fluid resuscitation should be prioritized in patients with sepsis.

### Clinical Update #1

Broad-spectrum antimicrobials (cefepime, metronidazole, and vancomycin) are initiated, and three NS boluses (each 20 ml/kg) are given followed by intravenous acetaminophen. A large diameter peripheral catheter is placed to ensure that fluid resuscitation is not delayed. After fluids and antibiotics, the child remains febrile with persistent tachycardia (HR 158) and hypotension (BP 70/30). Progressive tachypnea (RR 62) and oxygen requirement (5L vent mask) develop, and the child is transferred to the Pediatric Intensive Care Unit (PICU) for further management.

### What Additional Evaluations Should Be Undertaken at This Time?

The patient now meets criteria for septic shock, as evidenced by persistent hemodynamic dysfunction despite appropriate prompt fluid resuscitation. In addition to continuous assessment of vital signs, perfusion, and physical exam, ongoing evaluation of MOI should include repeat arterial blood gas (ABG), CBC, comprehensive metabolic panel, and coagulation studies. The patient should also have a chest radiograph to evaluate his new onset of respiratory distress. Further imaging may be warranted including computed tomography (CT) imaging given its increased sensitivity for detecting parenchymal disease in the context of neutropenia (14), but only after hemodynamic stability is achieved.

#### Take Home Message

After rapid resuscitation, comprehensive clinical reassessment is needed to determine response to therapy and to monitor for potential clinical deterioration.

### What Further Management Should Be Considered?

Treatment of septic shock should focus on improving oxygen delivery, reducing metabolic demand, and reversing the underlying trigger. Fluid resuscitation can augment cardiac output and therefore is a key method to improve oxygen delivery. Vascular access should not limit the ability to provide aggressive fluid resuscitation in a timely manner, and placement of peripheral intravascular catheters are often necessary. Many patients with septic shock may require more than 60 mL/kg of fluid resuscitation. Measuring intravascular volume status with physical exam, central venous pressure and respiratory variability in the arterial pulse pressure waveform can help differentiate patients who are adequately fluid resuscitated from those whom are not. While intravascular volume repletion is critically necessary, the capillary leak associated with myeloablative conditioning and sepsis may lead to pulmonary edema and anasarca.

Oxygen delivery can also be improved by providing supplemental oxygen (non-invasively or through endotracheal intubation) and increasing oxygen carrying capacity through RBC transfusions. In the absence of severe anemia or bleeding, transfusions should be used after initial crystalloid resuscitation. Oxygen delivery can also be augmented by improving cardiac output with vasoactive infusions. Pediatric HCT patients with sepsis may have hyperdynamic cardiac function, but concomitant myocardial depression due to prior anthracycline exposure, total body irradiation, or cytokine storm (15, 16) should prompt the use of vasoactive infusions (typically epinephrine or norepinephrine). Metabolic demand can be reduced with endotracheal intubation and sedation. Lastly, some pediatric HCT patients may have acquired adrenal insufficiency due to long-term exposure to glucocorticoids, so stress-dose hydrocortisone with a gastric acid protectant may be warranted.

Persistent sepsis should also warrant consideration for broadening antimicrobial therapy, particularly to provide coverage for MRSA and/or invasive fungal infection (IFI) like candidemia and molds, especially in the context of persistent fever and neutropenia (8). However, the presence of MOI will impact the type of empiric antimicrobial therapy given. For example, echinocandins or the triazole posaconazole can be administered in the context of hepatic and renal insufficiency (17), but interactions with immunomodulatory agents and potential for QTc prolongation should be noted with the azole agents (18). In contrast, vancomycin should be used with caution in the context of acute kidney injury (AKI) and dosed by trough if other alternative agents like linezolid are not available (19). Lastly, caution should be exercised with certain combinations of antimicrobial agents like vancomycin and piperacillin-tazobactam, which increases the risk of AKI (20).

#### Take Home Message

Individualized cardiopulmonary support and antimicrobial therapy are crucial in managing post-HCT septic shock.

### Clinical Update #2

In the PICU, CXR reveals bilateral pleural effusions (R > L) with increased interstitial markings. The child’s work of breathing increases and he becomes more lethargic, necessitating endotracheal intubation with mechanical ventilation. Given his continued hypotension (BP 75/28) and the high rate of peri-intubation cardiac arrest (21), he is also started on an epinephrine infusion (0.1 mcg/kg/min). Repeat labs show continued profound neutropenia (ANC < 200/μL), Hgb 7.2 g/dL, platelets 14 × 10^3^/μL, Cr 1.1 mg/dL, ALT 145 U/L, AST 179 U/L, INR 1.9, PTT 46 s (reference 24–36 s), fibrinogen 100 mg/dL (170–410 mg/dL), d-dimers 13.2 μg/mL (<0.5 μg/mL), lactate 5.6 mmol/L (<2.2 mmol/L), and procalcitonin 23 ng/ml (<5 ng/ml). RBC and platelet infusions are administered to support oxygen carrying capacity and to prevent bleeding. After hemodynamic stabilization, CT sinus, chest, abdomen and pelvis are performed. CT sinuses reveal opacification of the maxillary and ethmoid sinuses with air-fluid levels. CT chest shows opacification and air bronchograms in the left lower lobe with associated micronodules throughout both lungs and bilateral pleural effusions (R > L). CT abdomen/pelvis is negative for colitis or pneumatosis.

### How Can We Determine the Cause for This Patient’s Septic Shock?

Even in patients receiving broad-spectrum antimicrobial therapy, additional diagnostic microbiologic testing should be strongly considered in the setting of progressive septic shock and negative, indeterminate, or pending diagnostic tests. Standard clinical microbiologic diagnostics fail to identify a pathogenic organism in approximately one-third of suspected infectious cases (22) and delayed or missed microbial detection is associated with significant mortality (23–25). In addition to identifying an etiology for septic shock, diagnostic testing can identify plausible co-pathogens requiring treatment and determine antimicrobial susceptibilities, allow for cessation of unneeded nephro-, hepato-, and myelotoxic antimicrobials, and locate infected tissue that may require debridement for source control. Strategies for invasive testing should be guided by physical exam and imaging. Of note, chest x-ray alone has very poor sensitivity compared to chest CT in identifying potential pulmonary infections (26, 27). Bronchoalveolar lavage (BAL) can facilitate detection of pulmonary pathogens via direct sampling of infiltrates or consolidations when accessible (28) and particularly when performed early after symptom onset and radiographic confirmation (29). Lung biopsy should be reserved for lesions or pulmonary nodules not accessible by BAL, as complications associated with lung biopsy are greater than BAL, particularly in pediatric patients (30). Investigation should also consider the following: (a) rhinocerebral infection in patients with longstanding glucocorticoid use, neutropenia, or T-cell impairment, (b) colitis in patients with intestinal MBI or pre-transplant history of inflammatory bowel disease, (c) thrombophlebitis in patients with high-risk CVCs, (d) pyelonephritis in patients with urinary retention or indwelling catheters, (e) cellulitis in patients with pressure ulcers, (f) and cholecystitis in patients with chronic hemolysis. Novel testing methods using culture-independent techniques such as metagenomics sequencing have shown early utility in identifying pathogens in HCT and non-HCT patients with sepsis (31, 32).

#### Take Home Message

Pediatric HCT patients with septic shock should undergo extensive microbiologic testing in order to improve the likelihood of successful infectious diagnosis and treatment.

### Clinical Update #3

The following day, the patient undergoes bronchoalveolar lavage and samples are sent for culture, cytology, and PCR. ENT also performs nasal endoscopy and sinus samples are sent for culture and PCR. Preliminary gram stain of the BAL fluid shows numerous gram-negative rods. Blood cultures obtained at the time of initial fever remain negative. BAL and sinus cultures are positive for an extended-spectrum β-lactamase (ESBL)-producing *Pseudomonas aeruginosa*. Additional lab results include tacrolimus level was 17.2 ng/mL (reference 5–20 ng/mL), serum Aspergillus galactomannan (GMN) is 0.3 (positive >0.5). The patient continues to require vasopressors and remains profoundly neutropenic and transfusion dependent.

### What Further Management Is Required to Stabilize This Patient?

**FIGURE 2:**
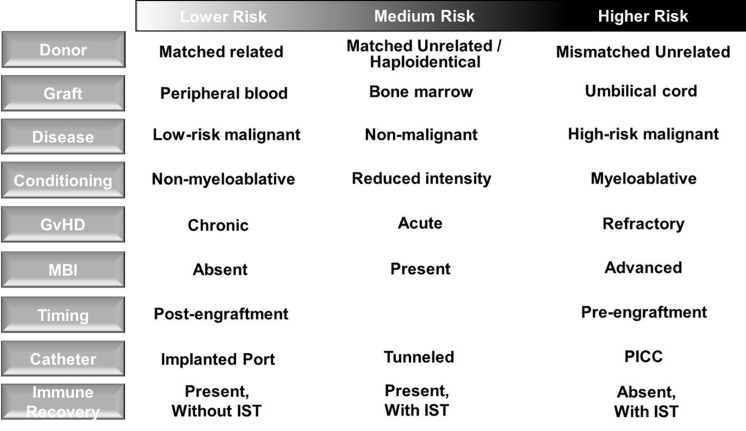
Risk factors for blood stream infection in allogeneic hematopoietic cell transplant recipients. **(Left column)** Transplant, donor, allograft and recipient factors are associated with risk for bloodstream infection (BSI). Higher risk patients should be monitored closely for the development of BSI in order to initiate prompt treatment aimed at preventing progression to severe sepsis or septic shock. GvHD, graft-versus-host disease; MBI, mucosal barrier injury; IST, immunosuppressive therapy; PICC, peripherally inserted central catheter.

Blood stream infections (BSI) are the most common infection following allogeneic HCT (33) and associated with the greatest mortality risk in HCT patients (34), particularly when MDR organisms are involved (35) and insufficient empiric antimicrobial therapy is administered (36). Attributable risk factors for BSI include presence of central venous catheter (CVC) placement including CVC type (37), MBI (38), and GvHD (39) as well as use of immunosuppressive therapy (IST) (40) (Figure 2). Pneumonia is also a common cause of infection after allogeneic HCT and associates with increased mortality in the first 100 days after transplant (41). Of note, HCT recipients often have multiple infections, which may be caused by different pathogens. Therefore, thorough diagnostic investigation and acquisition of diagnostics from sites of infection are warranted.

Once the pathogen is identified and antimicrobial sensitivities are defined, therapy targeting the specific pathogen(s) should be administered. However, additional considerations for the antimicrobial therapy include: location of the infection and the ability of the agent(s) to achieve adequate concentrations, presence of organ dysfunction affecting the metabolism of selected agent(s), drug interactions between antimicrobial and immunosuppressive therapies, and the clinical context in which a pathogen is identified and the antimicrobial therapy is administered. In this clinical case, the patient remains septic and with pancytopenia, requiring broad-spectrum antibiotic and antifungal therapies in addition to therapy targeting his ESBL-producing *Pseudomonas aeruginosa* (42). To this end, meropenem and caspofungin are started as empiric antimicrobial therapy.

In addition to administering proper antimicrobial therapy, other therapeutic interventions should be considered in this patient. First, removal of CVCs should be considered when there is evidence of refractory shock caused by MDR pathogens (43, 44). Second, salvage therapies aimed at restoring immunity should be considered and include reduction in or even withdrawal of immunosuppression, use of colony stimulating factors (G-CSF and GM-CSF) (45), and granulocyte transfusions (46). Augmenting immune recovery is key to best outcomes, but both cytokines and granulocyte transfusions have potential adverse effects like worsening inflammation, bone pain, and hypoxemia. Lastly, discontinuing medications with high toxicity profiles that are unlikely to help the patient may prevent further acute kidney injury (AKI). Other efforts to prevent or mitigate the development of organ toxicity should also be investigated. Pediatric HCT patients frequently receive multiple nephrotoxic medications, including antibiotics like glycopeptides and aminoglycosides, calcineurin inhibitors (e.g., tacrolimus, cyclosporine), and antivirals (e.g., acyclovir, foscarnet, ganciclovir, cidofovir). So early withdrawal or dose reductions in these medications to prevent advancing AKI is prudent. Other supportive care considerations to maintain organ function includes lung-protective mechanical ventilation, hemodynamic and transfusion support, thromboprophylaxis, careful fluid management, and sedation and analgesia.

#### Take Home Message

Reversing septic shock in pediatric HCT patients may require proper antimicrobial therapy, source control, and potential use of immunomodulatory therapies. Maintaining multiorgan function is critical to facilitate recovery.

### Clinical Update #4

After CVL line removal and appropriate antimicrobial therapy is administered, epinephrine is able to be weaned and ultimately discontinued on PICU day 4. The patient is extubated on PICU day 5 and transitioned to CPAP during the day and BiPAP at night for the next 3 days. The child begins dexmedetomidine and fentanyl weaning following extubation. Oxygen supplementation is weaned to 0.5L nasal cannula on PICU day 9. After a 10-day PICU stay, the child is transferred to the BMT unit to complete a 14-day course of antimicrobial therapy and to continue sedation wean. He received physical, occupational, and recreational therapies in the BMT unit. He has neutrophil engraftment (ANC 880/μl) on transplant day 21, and platelet engraftment on transplant day 37. He is discharged from the hospital on transplant day 42.

### What Is the Best Way to De-Escalate Therapy and Promote Recovery?

Pediatric HCT patients who survive septic shock may require weeks to recover from the immediate sequelae. Immunosuppression necessary for GvHD prophylaxis should be restarted and levels monitored in the context of continued but resolving organ dysfunction. Gradual reduction in sedation and analgesia is required to prevent withdrawal. A multimodal approach to prevent, detect, and treat delirium should be implemented (47). Long-term effects on renal, hepatic, and lung function should be monitored as survivors frequently have prolonged impairment (48). Lastly, many survivors have prolonged functional impairment that may benefit from rehabilitation services (49).

#### Take Home Message

Pediatric HCT patients who survive sepsis require continued supportive care to promote recovery, including monitoring for delirium, withdrawal and deconditioning.

### Patient #1 Summary

This case describes a pediatric HCT patient with fulminant bacterial septic shock due to resistant *Pseudomonas aeruginosa* prior to engraftment. Allogeneic HCT patients are at very high risk for bacterial sepsis in the pre-engraftment period (42). Prompt recognition, early empiric therapy, and hemodynamic resuscitation guided by physical exam and laboratory data are vital to ensuring survival (10). Chances for best clinical outcome require immune recovery, source control, and appropriate antimicrobial therapy (7). Duration of antimicrobial therapy is predicated upon overlap with immune recovery and documented eradication of infection by culture and/or radiographic resolution as clinically appropriate (50). Lastly, significant deconditioning associated with prolonged hospitalization, increases risk for infection and resource utilization, for which clinical therapy services to increase strength and stamina have been proven effective (51, 52).

## Clinical Vignette #2

### Initial Clinical Presentation

A 16-year-old female diagnosed with idiopathic severe aplastic anemia received an allogeneic BMT following fludarabine/cyclophosphamide/alemtuzumab (FCC) conditioning from a 12/12 HLA-matched unrelated donor 40 days ago. Her transplant course was unremarkable, and she was discharged on from the hospital on day +27 on tacrolimus as GvHD prophylaxis along with other supportive care medications including voriconazole, acyclovir and trimethoprim-sulfamethoxazole. She now presents to the emergency room with fever, hypotension, diffuse rash, cough and diarrhea. Sick contacts are notable at home with upper respiratory tract symptoms, including rhinorrhea and cough. Vital signs in the emergency department are as follows: 38.9°C, HR 116, RR 22, BP 74/45, SpO_2_ 95% on room air. Physical exam is significant for diffuse, blanching maculopapular rash involving torso, legs and arms sparing palms and soles, crackles mostly in the bilateral bases, and mild abdominal tenderness with palpation. Her double-lumen tunneled catheter site is without erythema, discharge or tenderness. Blood cultures are obtained from both catheter lumens, and respiratory viral PCR, CBC and metabolic panels are sent. CXR is obtained that demonstrates bilateral interstitial markings. Two NS fluid boluses are given, and empiric antimicrobial therapy with piperacillin-tazobactam is started.

### What Additional Clinical and Laboratory Information Will Be Useful?

This patient meets all SIRS criteria and is at high-risk for infection without a clear alternate etiology; therefore, the diagnosis of sepsis should be suspected. Although this patient has achieved some level of innate immunity due to being post-engraftment, adaptive immunodeficiency persists through at least the first year post-HCT and is compounded by ongoing immunosuppression from GVHD prophylaxis, placing engrafted pediatric HCT patients at risk for sepsis (Figure 1). The differential diagnosis for the type of sepsis remains broad; so bacterial, viral, and fungal etiologies should be investigated. Comprehensively staging the patient’s organ dysfunction is needed both to determine if the patient meets criteria for severe sepsis and to anticipate upcoming need for multiorgan support. Because the etiology of this patient’s abrupt decline is not clear at this time, other potential diagnoses, including acute GvHD and hemophagocytic lymphohistiocytosis (HLH), should be considered, particularly if the patient remains critically ill and initial diagnostic work-up is negative, indeterminate, or pending.

#### Take Home Message

A pediatric HCT patient with fever and hypotension without a clear etiology merits rapid comprehensive diagnostic evaluation and intervention.

### How Should This Patient Be Managed?

Fluid resuscitation should be guided by physical exam and laboratory data. Specifically, this patient’s persistent tachycardia and hypotension without evidence of acute heart failure (i.e., peripheral edema and hepatomegaly) suggest ongoing need for volume expansion. As with the patient in clinical vignette #1, oxygen delivery can be improved through supplemental oxygen and vasoactive infusions. Empiric antimicrobials should be initiated promptly and include a broad-spectrum antimicrobial like piperacillin-tazobactam or cefepime. Gram-positive coverage would be warranted if the patient has a focus of infection (e.g., catheter site soft tissue/skin infection) or continues to be hemodynamically unstable. Broader empiric antifungal coverage would similarly be considered for continued clinical instability.

#### Take Home Message

All pediatric HCT patients with suspected sepsis, severe sepsis, or septic shock should receive prompt fluid resuscitation and empiric antibiotics while diagnostic evaluation is ongoing.

### Clinical Update #1

Initial laboratory results are as follows: WBC 15.9 × 10^3^/μL, ANC 12,250, ALC 189, Hgb 12.1 g/dL, platelets 95 × 10^3^/μL, Cr 2.6 mg/dL (reference 0.6–1 mg/dL), ALT 452 U/L, AST 675 U/L, and total bilirubin 3.4 mg/dL, direct bilirubin 3.2 mg/dL, CRP 24.9 mg/dL (reference <1.2 mg/dL), lactate 5.7 (0.5–2.2 mmol/L), procalcitonin 5 ng/mL (<0.5 ng/mL). While still in the emergency department, the patient receives two more NS boluses due to persistent hypotension (BP 60/30) and is started on epinephrine (0.1 mcg/kg/min) prior to transfer to the PICU.

### Do These Findings Change This Patient’s Risk Stratification?

Initial laboratory findings show significant multiorgan injury (MOI). Assuming a baseline creatinine of <1 mg/dL, this patient’s Cr has more than doubled, suggesting greater than 50% reduction in glomerular filtration and sufficient to already qualify for a diagnosis of AKI (53). In addition, the patient demonstrates transaminitis consistent with liver injury as well as an elevated lactate suggesting end-organ dysfunction. Persistence of cardiovascular dysfunction refractory to fluid resuscitation qualifies this patient for a diagnosis of septic shock. RBC transfusions are not indicated with a hemoglobin of 12 g/dL.

#### Take Home Message

This patient met criteria for severe sepsis on presentation and quickly escalated to fluid-refractory septic shock.

### Clinical Update #2

In the PICU, the patient develops tachypnea and hypoxemia, for which she is placed on 10L ventilation mask. Repeat CXR reveals bilateral pleural effusions with persistent interstitial markings. Given hypercapnia and continued tachypnea with persistent work of breathing, she is intubated and positive pressure ventilation is initiated. In addition to respiratory failure, the patient continues to have significant hypotension (BP 65/30) despite epinephrine. Therefore, norepinephrine is started (0.2 mcg/kg/min) and hydrocortisone 100 mg/m^2^ is administered for presumed adrenal insufficiency.

The patient is noted to have bloody diarrhea and frank blood is suctioned from her endotracheal tube (ETT). Coagulation studies reveal INR 3.2, PTT 60 s, and fibrinogen 58 mg/dL. GI film array is negative. Other labs are as follows: ferritin 580 ng/mL (reference 31–294 ng/mL), triglycerides 190 mg/dL (29–200 mg/dL), tacrolimus 28.2 ng/mL (5–20 ng/mL) and serum Aspergillus galactomannan (GMN) 0.4 (positive >0.5).

### What Diagnostic Management Considerations Are Required at This Point?

Fulminant respiratory failure can frequently occur in the setting of post-HCT sepsis. It may not be initially clear if the lungs are the primary source for the sepsis (direct lung injury) or have become injured as sequelae of sepsis (indirect lung injury). Prompt endotracheal intubation with aggressive attempts to maintain functional residual capacity through adequate mean airway pressure may be required in the face of ongoing loss of pulmonary compliance. Pediatric HCT patients with respiratory failure frequently meet criteria for acute respiratory distress syndrome (ARDS), which is associated with >60% mortality in this population (54). Pulmonary hemorrhage is a disastrous complication of post-HCT lung injury and requires prompt restoration of hemostasis. Intravenous or inhaled anti-fibrinolytics (e.g., tranexemic acid) or clotting factors (e.g., recombinant factor VII) may be administered for short term hemostasis while the underlying etiology is addressed. Adrenal insufficiency should be considered in patients with vasopressor-refractory shock and echocardiography should be used to assess myocardial dysfunction. Strategies to reduce metabolic demand should be promptly employed, including sedation, paralysis, cessation of enteral feeding, and anti-pyresis. Nephrotoxic medications like tacrolimus should be discontinued to prevent further renal injury and levels should be followed daily to determine when dosing can be safely resumed. While supportive care is ongoing, additional diagnostics should include broad assessment for viral infection, including qualitative PCR from plasma, stool, nasal, and ETT aspirate for adenovirus, cytomegalovirus, herpes simplex virus, and other herpesviruses. Reflex plasma quantitative PCR should be obtained if qualitative PCR is positive.

#### Take Home Message

Pediatric HCT patients with septic shock and MOI require aggressive intensive care aimed at stabilizing organ function, which should occur in parallel with an exhaustive diagnostic work-up to identify underlying etiology and to direct treatment.

### Clinical Update #3

Blood cultures and plasma qualitative PCR for CMV, EBV, and HHV-6 are negative. However, qualitative adenovirus (ADV) PCR from urine, stool, and ETT secretions are positive; and reflex quantitative plasma ADV PCR shows 757,630 DNA copies/mL. Skin biopsy obtained after correction of systemic coagulopathy is negative for GvHD, but qualitative PCR is positive for ADV. However, repeat laboratory tests show persistent lactic acidosis and further elevation in creatinine to 3.5 mg/dL.

### How Can Disseminated Adenovirus Be Effectively Treated in This Patient?

**FIGURE 3:**
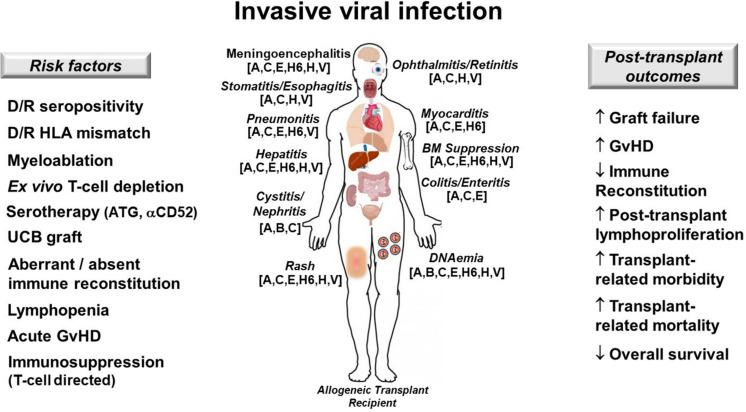
Invasive viral infection in allogeneic hematopoietic cell transplant recipients. Risk factors **(left)**, target organs and clinical manifestations **(center)** and post-transplant outcomes affected by DNA viruses **(right)** are depicted. Patients at risk for dsDNA viremia should be monitored closely for the development of DNA reactivation or *de novo* infection and should be treated promptly in order to prevent progression to severe multi-organ injury (MOI). A, ADV, adenovirus; αCD52, anti-CD52 monoclonal antibody (alemtuzumab); ATG, antithymocyte globulin; B, BK virus; C, CMV, cytomegalovirus; D/R, donor/recipient; E, EBV, Epstein-Barr virus; GvHD, graft-versus-host disease; H6, HHV-6, human herpesvirus 6; H, HSV, herpes simplex virus; HLA, human leukocyte antigen; UCB, umbilical cord blood; V, VZV, varicella zoster virus; ↑, increases; ↓, decreases.

Disseminated adenovirus (ADV) is a cause of viral sepsis that can quickly escalate to MOI and death following allogeneic HCT, especially in the context of either high ADV loads (55) or co-infection with other double-stranded DNA viruses (56). ADV can cause myocarditis, pneumonitis, cystitis, hepatitis, enteritis, and meningoencephalitis in the immunocompromised host (57) (Figure 3).

The patient has numerous risk-factors for presumed ADV reactivation (as pre-transplant serology was not performed), including alemtuzumab-based serotherapy, recent lymphocyte reconstitution, and ongoing T-cell targeted immunosuppression (58) (Figure 3). Treatment of disseminated ADV includes intravenous cidofovir and rapid tapering of immunosuppression (59). Novel investigational therapies for disseminated adenovirema include virus-specific T-cells (VSTs), which are available “off the shelf” from unrelated donors or generated from a related donor (60). As the patient is greater than 28 days from serotherapy, does not have GvHD, and is not receiving steroids, consideration for VSTs is reasonable at this time.

Since obtaining VSTs may take time depending upon how they are manufactured (61), organ function preservation is crucial while potential therapies are mobilized. Specifically, the patient has significant AKI that would likely be worsened by intravenous cidofovir at standard dosing (5 mg/kg/week) despite the use of probenecid. Therefore, the patient may be a candidate for thrice weekly cidofovir at reduced dosing (1 mg/kg/dose) and continuous veno-venous hemodialysis (CVVHD) in order to maintain extracorporeal renal function and facilitate fluid removal while salvage cell therapies are initiated.

#### Take Home Message

Disseminated viral infections in pediatric HCT patients can cause MOI and requires aggressive antiviral and consideration for experimental therapies. Strategies to treat post-HCT viral infections are constantly evolving and BMT, ICU, and ID physicians should work collaboratively to obtain investigational therapies for their patients.

### Clinical Update #4

On PICU day 4, the patient is started on cidofovir 1 mg/kg/dose thrice weekly. Given the severity of disease and renal insufficiency, the patient undergoes placement of a dialysis catheter and receives CVVHD. She receives haploidentical ADV-specific T-cells enriched using a commercial cytokine capture system via eIND on PICU day 10. The patient’s quantitative plasma ADV PCR decreases to 15,250 and 585 DNA copies/mL 7 and 10 days after VST infusion, respectively. She is extubated on PICU day 22 and ultimately transferred to the BMT floor on hospital day 25 while supplemental oxygen via 2L nasal cannula and undergoing sedation wean.

### With Adenovirus Responding to VST Therapy, What Future Care Considerations Are Needed?

After two consecutive plasma qualitative ADV PCR separated by 72 h are negative, antiviral pharmaceutical therapies can be discontinued, but close monitoring is needed for signs of viral recrudescence, including serial plasma and stool ADV PCR, end organ laboratory monitoring, and close physical exam. In addition, patients with dsDNA viral infections after HCT are at high risk for delayed IR (62), bone marrow suppression and potential development of gastrointestinal GvHD (63). This patient should be followed closely for these complications while re-initiating GVHD prophylaxis and monitoring for organ dysfunction.

### Patient #2 Summary

This clinical case describes a pediatric HCT patient with fulminant viral septic shock in the setting of incomplete lymphocyte functional reconstitution. Pediatric HCT patients are at high-risk for viral syndromes including viremia, end organ infection (pneumonitis, enteritis, and meningoencephalitis) and disseminated sepsis. Prompt recognition, early empiric therapy, and hemodynamic resuscitation guided by physical exam and laboratory data are crucial to ensuring survival.

## Clinical Vignette #3

### Initial Clinical Presentation

An 8-year-old boy with relapsed T-cell acute lymphocytic (T-ALL) received a 10/10 HLA-matched unrelated PBSC transplant 67 days ago using cyclophosphamide/total body irradiation (TBI) conditioning and methotrexate/tacrolimus GvHD prophylaxis. He is receiving oral voriconazole, acyclovir and trimethoprim-sulfamethoxazole as antimicrobial prophylaxis. He is currently admitted for severe (Grade 3), skin (Stage 3) and gastrointestinal (Stage 2) GvHD. For his GvHD, he was started on 2 mg/kg methyl-prednisolone. However, after 5 days of steroids, his GvHD was deemed steroid-refractory (SR). Therefore, he was started on infliximab (64) and remains on a weaning dose of steroid and tacrolimus. On hospital day 17, he has a fever and vomiting and complains of nausea and abdominal pain. Vital signs are as follows: 39.9°C, HR 159, RR 28, BP 62/30, SpO_2_ 95% on room air. Physical exam is significant for white plaques on his buccal mucosa, diffuse maculopapular rash involving torso, legs and arms and mild abdominal tenderness with deep palpation. His double-lumen tunneled catheter site is without erythema, discharge or tenderness. Blood cultures are obtained from both catheter lumens, CBC and metabolic panels are sent. CXR is obtained that demonstrates no acute cardiopulmonary process. Two NS fluid boluses are given. He is started on empiric vancomycin and meropenem and transferred to the PICU for further management.

### What Additional Clinical Considerations Arise?

Acute GvHD is a significant risk factor for infection and infection-related mortality in allogeneic HCT patients (65). In particular, risk for invasive fungal infection (IFI) is especially high in patients with SR GvHD (66), likely reflecting underlying pathophysiology and use of immunomodulatory agents that hinder donor-derived antifungal immunity. Such increased risk in infection even occurs with the use of newer immune therapies for SR GvHD like ruxolitinib (67). While the patient had been receiving voriconazole prophylaxis, he is at significant risk for break-through IFI given his SR gastrointestinal GvHD and is hemodynamically unstable. Therefore, consideration for starting empiric liposomal amphotericin is warranted.

#### Take Home Message

Graft-versus-host disease and its associated therapy significantly increase risk for IFI in allogeneic HCT recipients by compromising antimicrobial immunity and damaging mucosal barrier integrity, enabling transmigration of gut flora into the blood stream as well as hindering absorption of oral antifungal prophylaxis.

### Clinical Update #1

Initial laboratory results are as follows: WBC 23 × 10^3^/μL, ANC 18,850, ALC 120, Hgb 10.1 g/dL, platelets 65 × 10^3^/μL, Cr 2.2 mg/dL (reference 0.6–1 mg/dL), ALT 78 U/L, AST 90 U/L, total bilirubin 1.2 mg/dL, direct bilirubin 0.6 mg/dL, CRP 6 mg/dL (reference <1.2 mg/dL), lactate 2.3 (0.5–2.2 mmol/L), procalcitonin 7.8 ng/mL (<0.5 ng/mL), beta-D-glucan 80 pg/mL (>60 pg/mL). The patient receives two additional NS boluses due to persistent hypotension (BP 62/35) and is started on epinephrine (0.1 mcg/kg/min). He is started on liposomal amphotericin, and his previous antibiotic therapies are maintained.

### How Reliable Are Serum Biomarkers for Diagnosis of IFI in Pediatric HCT Recipients?

Proven diagnosis of IFI requires isolation of the fungus from a sterile site in a patient suspected of having a fungal infection (68). Fungal biomarkers like galactomannan (GMN) and (1,3)-beta-D-glucan (BDG) can aid in the diagnosis of probable IFI, but alone lack the definitive diagnostic capability given their variability in sensitivity, specificity and positive predictive value in the clinical setting of pediatric HCT (69).

### Clinical Update #2

Nine hours after collection, blood cultures from both catheter lumens are reported positive for yeast. The isolate is ultimately identified as *Candida parapsilosis*, and susceptibility testing shows the strain to be sensitive to voriconazole, posaconazole, and amphotericin. The patient’s central line is removed within 24 h after the blood cultures were positive for yeast, and both catheter lumens growing *C. parapsilosis*. The patient remained hemodynamically stable and is ultimately weaned off his epinephrine on PICU day 3. However, he remains febrile despite line removal and persistent negative daily blood cultures.

### What Is the Diagnostic Work-Up for Fungemia in an Immunocompromised Patient?

**FIGURE 4:**
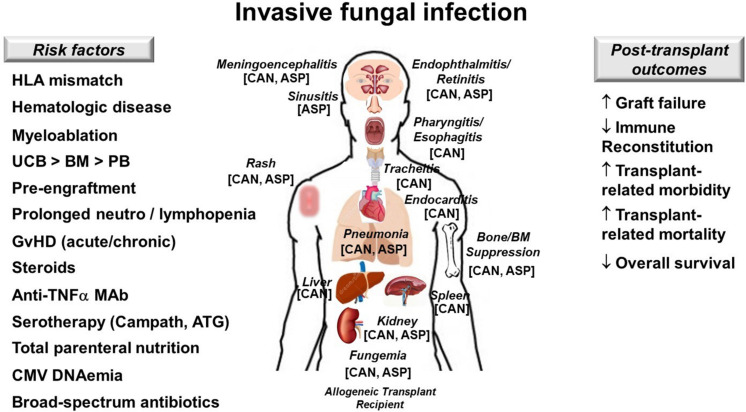
Invasive fungal infection in allogeneic hematopoietic cell transplant recipients. Risk factors **(left)**, target organs and clinical manifestations **(center)** and post-transplant outcomes affected by representative fungi **(right)** are illustrated. Keys to best outcomes following IFI include adequate source control, proper antifungal therapy and most importantly restoration in antifungal immunity. ASP, *Aspergillus* spp.; ATG, antithymocyte globulin; BM, bone marrow; CAN, *Candida* spp.; GvHD; graft-versus-host disease; HLA, human leukocyte antigen; MAb, monoclonal antibody; PB, peripheral blood; UCB, umbilical cord blood; ↑, increases; ↓, decreases.

An immunocompromised patient with candidemia warrants a work-up for invasive candidiasis (70, 71), particularly an allogeneic HCT recipient with SR GvHD with recent candidemia and persistent fever (Figure 4). The work-up involves ocular examination, echocardiogram and liver/spleen imaging to determine if the patient has retinitis, endocarditis, or hepatosplenic candidiasis, respectively. Other diagnostic considerations include lumbar puncture, chest imaging, and endoscopy if the patient has signs or symptoms of meningoencephalitis, pneumonia, or esophagitis, respectively. Urine culture and imaging of the kidneys should also be considered.

#### Take Home Message

Fungemia and persistent fever in an immunocompromised patient necessitates further diagnostic work-up to identify potential source for infection.

### Clinical Update #3

The patient’s transthoracic echocardiogram and ophthalmology exam are negative for infection. In addition, CT sinus and chest are also negative. However, CT abdomen/pelvis reveal multiple hypodense lesions in the liver and spleen consistent with hepatosplenic candidiasis. The patient is transferred back to the BMT unit where he is maintained on liposomal amphotericin until improvement in his GvHD, after which time he is transitioned to oral voriconazole and has therapeutic voriconazole levels confirmed (72).

### Patient #3 Summary

This case illustrates a pediatric HCT patient with SR aGvHD, which predisposes him to IFI manifesting as candidemia and hepatosplenic candidiasis. Breakthrough IFI can occur despite antifungal prophylaxis, especially in the setting of malabsorption causing sub-therapeutic levels. In addition to source control through the removal of central venous catheter, determination for other potential sites of fungal infection was needed. Outcomes for IFI in immunocompromised patients are most favorable when source control is achieved, proper antifungal therapy is administered, and immune recovery occurs.

## Conclusion

Pediatric allogeneic HCT patients who present with fever and hypotension are at high risk of developing sepsis from a variety of organisms with progression to septic shock. Early individualized multiorgan support is critical to prevent the progression of organ injury. Broad diagnostic testing is crucial so that tailored anti-pathogen therapies can be implemented before irreversible MOI develops. Ultimately, close collaboration between transplant, intensive care, and infectious disease teams remains a cornerstone of successful patient outcomes.

## Author Contributions

MZ, CD, and JA wrote and revised the manuscript. JA created the figures and organized the manuscript. All authors contributed to the article and approved the submitted version.

## Conflict of Interest

The authors declare that the research was conducted in the absence of any commercial or financial relationships that could be construed as a potential conflict of interest.
